# Improving Patient Safety and Patient–Provider Communication

**DOI:** 10.1044/2019_persp-19-00134

**Published:** 2019-10-31

**Authors:** Richard R. Hurtig, Rebecca M. Alper, Karen N. T. Bryant, Krista R. Davidson, Chelsea Bilskemper

**Affiliations:** a Department of Communication Sciences and Disorders, University of Iowa, Iowa City; b Department of Communication Sciences and Disorders, Temple University, Philadelphia, PA; c Voxello, Coralville, IA

## Abstract

**Purpose:**

Many hospitalized patients experience barriers to effective patient–provider communication that can negatively impact their care. These barriers include difficulty physically accessing the nurse call system, communicating about pain and other needs, or both. For many patients, these barriers are a result of their admitting condition and not of an underlying chronic disability. Speech-language pathologists have begun to address patients’ short-term communication needs with an array of augmentative and alternative communication (AAC) strategies.

**Method:**

This study used a between-groups experimental design to evaluate the impact of providing patients with AAC systems so that they could summon help and communicate with their nurses. The study examined patients’ and nurses’ perceptions of the patients’ ability to summon help and effectively communicate with caregivers.

**Results:**

Patients who could summon their nurses and effectively communicate—with or without AAC—had significantly more favorable perceptions than those who could not.

**Conclusions:**

This study suggests that AAC can be successfully used in acute care settings to help patients overcome access and communication barriers. Working with other members of the health care team is essential to building a “culture of communication” in acute care settings.

**Supplemental Material:**

https://doi.org/10.23641/asha.9990962

Hospitalization is often a stressful experience for patients and their families. Patients may be dependent on their health care providers for treatment and assistance with activities of daily living. Many patients require assistance from their nurses for everything from getting up to feeding and toileting. Being able to summon caregivers and effectively communicate is an essential part of best practices in the care of hospitalized patients. The design standards for hospitals require that each patient room be connected to a call system to enable patients to summon help (Facility Guidelines Institute, 2018). Typically, each bed is equipped with a call pendant that patients can activate to summon their nurses. The call pendants require manual dexterity and strength to operate—this can limit their use by physically impaired patients.

Zubow and Hurtig (2013) reported that 33% of conscious patients in intensive care units are unable to use the conventional nurse call systems. When a patient activates the call pendant, someone at the nursing station will typically respond remotely, ask the patient what they need, and then send someone to the bedside. The effectiveness of the intercom system relies on the patient’s ability to hear the message from the nursing station and to respond. Unfortunately, many patients experience barriers that render them unable to effectively respond. These barriers may be due to a hearing loss, mechanical ventilation, a motor–speech impairment, or a language barrier. Newer call systems allow the patient to directly communicate with their nurses’ smartphones. These systems enable patients to do more than simply call for help by letting them indicate what they need (e.g., pain, toileting). However, the new systems do not circumvent all of the communication barriers. Mechanical ventilation precludes patients from being able to respond to a query from the nurse and to speak with their caregivers. The estimates of the number of ventilated patients who are in need of assistance with communication range from 33% (Zubow & Hurtig, 2013) to 50% (Happ et al., 2015).

Poor patient–provider communication has been associated with an increased risk of experiencing an adverse (medical) event (AE; Agency for Healthcare Research and Quality, 2013a, 2013b; Bartlett, Blais, Tamblyn, Clermont, & MacGibbon, 2008; Cohen, Rivara, Marcuse, McPhillips, & Davis, 2005; The Joint Commission, 2010). Preventable AEs represent a huge problem for the U.S. health care system (Kohn, Corrigan, & Donaldson, 2000; Landrigan et al., 2010; Levinson, 2010). Estimates of the annual costs associated with the AEs that can result from poor patient–provider communication (adverse drug reactions, falls, pressure ulcers, and ventilator-associated pneumonia) have been estimated to exceed $29 billion (Hurtig, Alper, & Berkowitz, 2018). The costs associated with preventable AEs must be borne by hospitals. Furthermore, patient–caregiver communication barriers have been associated with increased patient stress and decreased patient satisfaction with care (Balandin, Hemsley, Sigafoos, & Green, 2007; Hemsley, Balandin, & Togher, 2007; Hemsley, Balandin, & Worrall, 2011; Hoffman et al., 2005; Rodriguez et al., 2016). Patient–provider communication now also plays a role in how hospitals are evaluated and compensated (Centers for Medicare and Medicaid Services, 2017).

To help prevent AEs, patients who cannot independently summon help require increased nurse vigilance and more frequent checks. Caring for patients who face communication barriers is stressful and time consuming. This then limits the time available to care for the other patients in the nurse’s caseload. Supporting patient communication and engagement with care is essential to maximizing nurse efficiency, interacting with physicians, and improving outcomes.

Numerous studies have described the positive relationship between patient–provider communication and health outcomes (Balandin et al., 2007; Blackstone, Beukelman, & Yorkston, 2015; Cohen et al., 2005; Costello, 2000; Divi, Koss, Schmaltz, & Loeb, 2007; Dowden, Honsinger, & Beukelman, 1986; Downey & Hurtig, 2006; Hemsley et al., 2007, 2011; Hurtig & Downey, 2009; Hoffman et al., 2005; Patak, Gawlinski, Fung, Doering, & Berg, 2004). Specifically, these studies showed that effective patient–provider communication is essential to health care access, participation in decision making, and the quality of care received. For example, patients need to communicate with their providers about new or changing symptoms to prevent unnecessary AEs. Patients also have a right to be involved in critical decision making about highly personal choices such as end-of-life issues.

There are ethical and financial reasons why health care institutions must address the significant number of AEs associated with communication barriers (Hurtig et al., 2018). A patient’s right to effective communication is laid out in the Joint Commission’s (2010) hospital accreditation standards. Meeting the needs of patients who face communication barriers clearly falls within the scope of practice of speech-language pathologists (American Speech-Language-Hearing Association, 2016, n.d.). Although there is a well-established positive relationship between patient–provider communication and health care outcomes, no large-scale studies assess the impact of implementing augmentative and alternative communication (AAC) strategies to support communication in the health care setting.

## Method

### Research Questions

1. Do patients’ perceptions of their ability to summon help and communicate vary as a function of their access to the nurse call system?
2. Is the AAC intervention perceived favorably by patients and their nurses?

### Subject Population

The study used a convenience sample of patients admitted to the hospital’s intensive care and step-down units. This study did not include patients with pre-existing cognitive or language impairments and patients whose admitting diagnosis involved a newly acquired cognitive or language impairment. The study was designed to compare three groups of patients: (a) patients using the Voxello technology due to their inability to independently access the nurse call (*noddle* group); (b) patients who could summon their nurses and effectively communicate without AAC (*full*-*access* group); and (c) patients who, prior to discharge, were unable to independently access the nurse call or communicate with their nurses (*no-access* group). The demographic data on the patients enrolled in the study are provided in Table 1. Due to ethical considerations, data were collected from the patients in the full-access and no-access groups, prior to the implementation of the Voxello technology at the hospital. The study protocol was reviewed and approved by the university’s institutional review board, and informed consent was obtained from all study participants. As part of the informed consent process, patients were provided with a full description of the purpose of the study and told that they would be asked to complete a survey about their perceptions of being able to summon help and communicate with their caregivers. The nurses who cared for the patients in the noddle group were also enrolled as study participants and asked to complete a survey about their patients’ ability to summon help and communicate with them.

## Intervention

**Figure 1.**
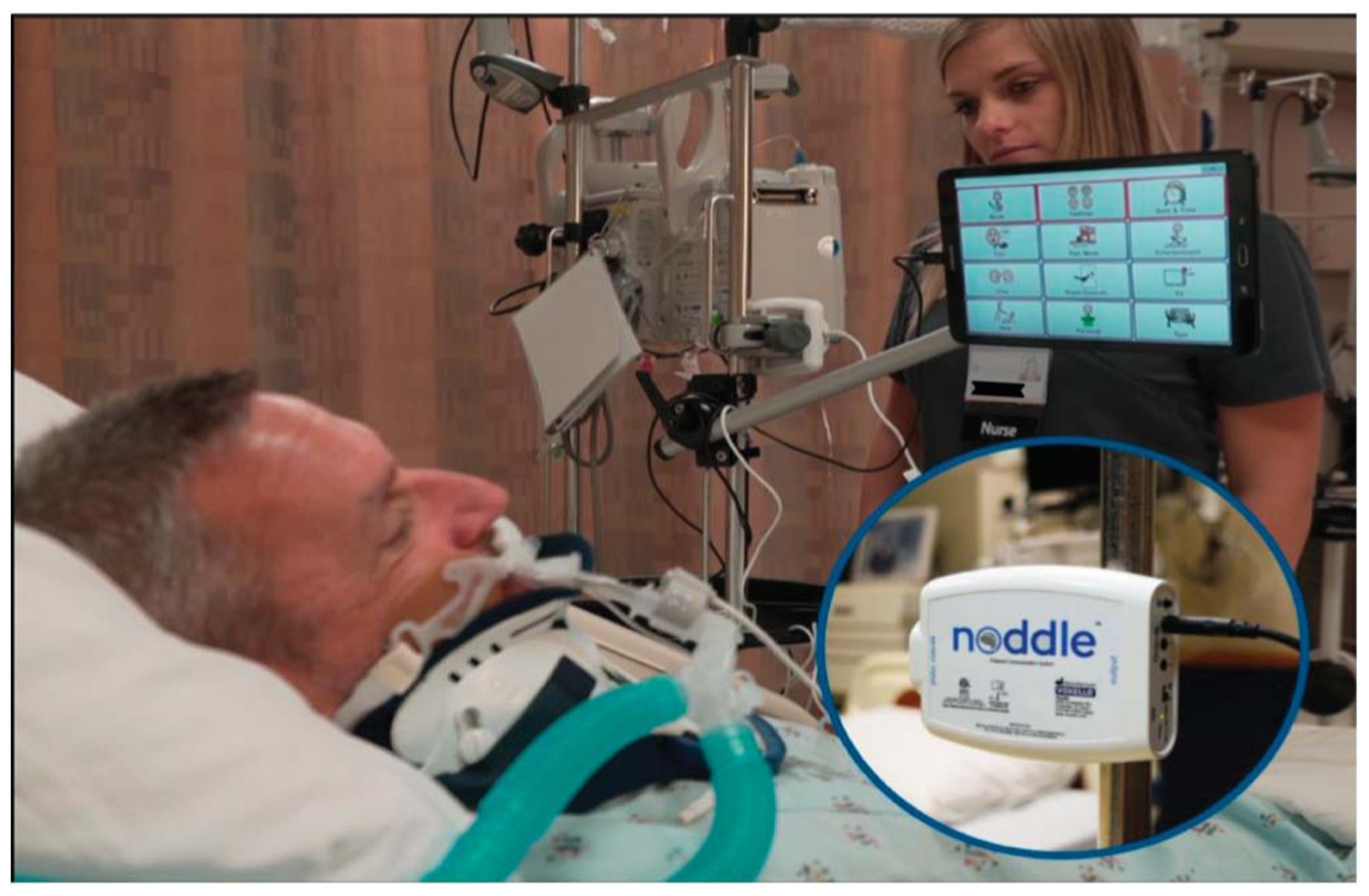
Demo of noddle and noddle-chat mounted on an intravenous pole at the bedside. Image used with permission.

**Figure 2.**
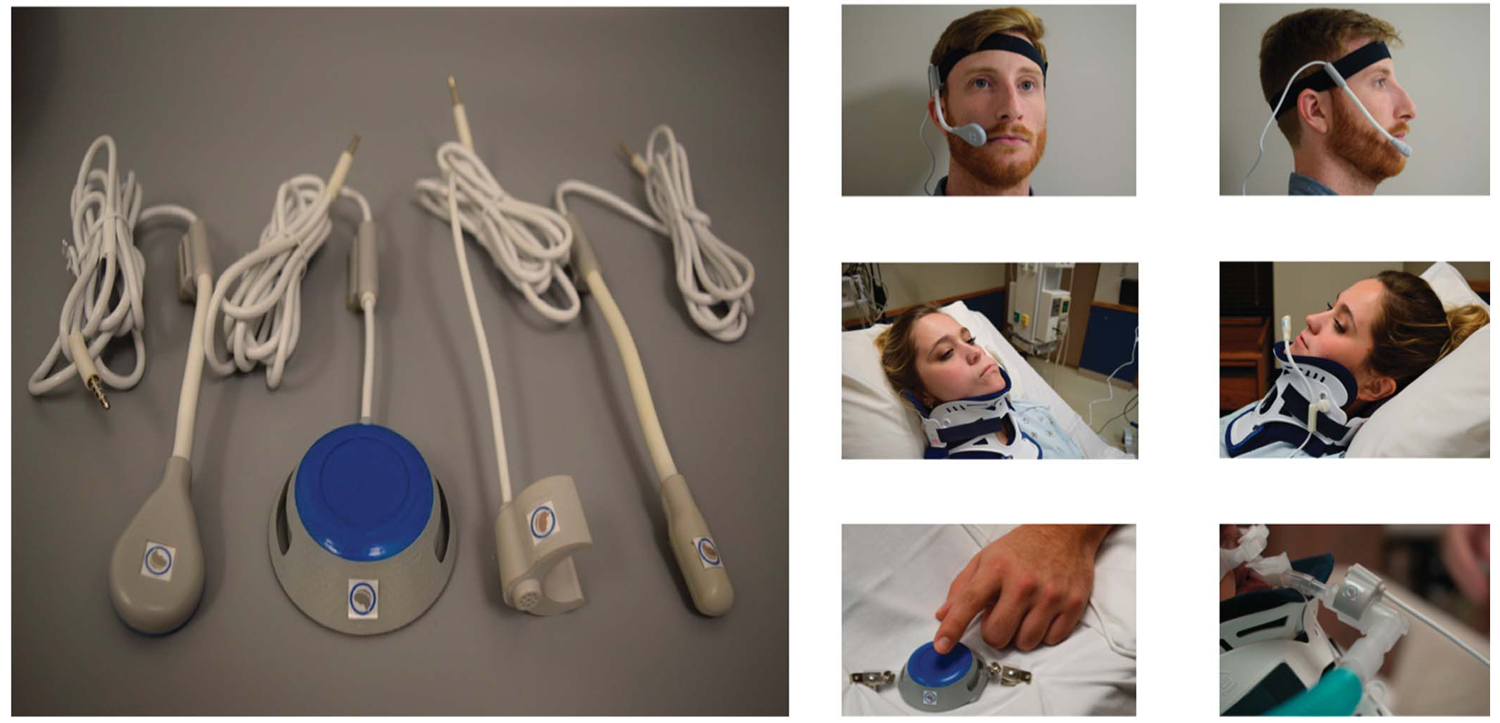
Noddle touch and microphone sensors and illustrations of sensor mounting options. Images used with permission.

The current study was specifically designed to assess the impact of providing an AAC intervention to overcome patients’ barriers to use of the nurse call system and to communicate with their nurses. Specifically, the study evaluated the impact of implementing the noddle switch and the noddle-chat communication tablet developed by Voxello in a large midwest teaching hospital. Figure 1 shows how the technology is mounted on a bedside intravenous pole. The Voxello technology was designed to enable patients to summon help when they could not use the standard nurse call pendant or standard Curbell alternatives such as the soft touch or pressure bulb. The noddle uses a gesture detection algorithm to allow patients to control the nurse call system and a speech-generating device (SGD). The technology can be used by patients who may only be able to produce a small intentional gesture (e.g., tongue click, head nod, shoulder shrug, finger tap). In Figure 1, the noddle receives input from the noddle vent mic that is clipped onto the vent line. Voxello has developed a set of sensors to be used with the noddle (see Figure 2). These sensors can respond to either small low-force movement gestures (J-touch and Bed-touch) or small tongue clicks (J-Mic and Vent-Mic). Additionally, the noddle can count the number of sequential gestures a patient produces. Based on the number of sequential gestures produced, a patient can use an SGD in two-switch scanning mode and activate the nurse call system. For example, with a single gesture, the patient can step through options on the SGD and, with a sequence of two gestures, select the particular option. A sequence of three gestures can be used to activate the nurse call system. As illustrated in the demonstration clip in (Supplemental Material) patients who may only be able to produce a single intentional gesture can use that gesture as if they had access to multiple switches. The noddle-chat application provided patients with multiple pages of preprogrammed messages and an on-screen keyboard to generate novel utterances (additional demonstration videos of how the system functions can be found at https://voxello.com/education-training/).

Unit nurses identified patients who were unable to access the nurse call system and who were unable to speak due to mechanical ventilation for participation in the noddle study. For the patients in the control groups, nurses identified patients who, at the end of their hospital stay, either had not had problems accessing the nurse call system and communicating with their nurses (full-access group) or were unable to access the nurse call and communicate with their nurses (no-access group). Members of the research team described the study and obtained informed consent from participants in the control groups who then were asked to complete the study survey (see Table 2). For patients in the noddle intervention groups, members of the research team determined whether the referred patients met the following inclusion criteria:

1. Consciousness status
  - The patient is able to respond to verbal or visual input.
  - The patient can produce an intentional gesture.
  - The patient can respond appropriately to simple yes/no questions.
  - The patient can understand that the intentional gesture can be used to call the nurse or control another device.
2. Sensory status
  - Vision: While it is optimal if the patient can see the speech-generating tablet, it is not required (the tablet can be used in an auditory scanning mode).
  - Hearing: The patient should be able to hear the communication partner and the output of the SGD (note: the tablet can display the message text).
  - Somatosensory: Only the noddle touch sensor may require sensory feedback if used with hand or finger movements.
3. Motor status: for direct select
  - Patients who can extend an arm and a finger, or hold a stylus, will be able to use the speech-generating tablet’s touch screen to directly select messages.
4. Motor status: for indirect select
  - The patient should be able to produce an intentional gesture.
    - Determine which gestures a patient can produce (e.g., tongue click, tongue in cheek, finger tap, head nod, shoulder shrug, wrist rotation).
    - Determine which gesture a patient prefers to use.
  - The patient needs to be able to produce a sequence of the intentional gestures to be able to control both the nurse call and the speech-generating tablet.
5. Sedation status
  - The patient should not be sedated while using the noddle, though patients who are unsedated for periods of time each day can use the technology when not sedated. Care should be used to reorient the patient to the use of the device each time sedation is withdrawn as some medications are known to impact memory.
6. Cognitive status for minimal use
  - Produce voluntary gesture on command.
  - Remember that voluntary gesture can be used to summon help.
7. Cognitive status for full use
  - Produce sequences of voluntary gestures.
  - Remember the function of specific gesture sequences (e.g., call nurse, use switch scanning with the speech-generating tablet).
  - Understand the message options on the tablet and how to navigate to the desired message. Note that message buttons have both text labels and symbols, so literacy is not required.
8. Language status
  - The patient should understand simple utterances with visual support if needed.
  - The patient should understand the link between message button label/symbol and the message that is associated with it.

For patients who met the inclusion criteria and who consented to participate in the study, members of the research team selected the appropriate noddle sensor (see Figure 2) and instructed the patient on how to use the voluntary gesture to control the nurse call system and the noddle-chat SGD. Members of the research team, working with the nursing department, also provided in-service training to unit nursing staff on use of the noddle switch and the noddle-chat SGD. To promote intervention fidelity, nurses and family members—when available—were provided just-in-time training on how to support the patient’s use of the technology.

## Results

### Data Analysis

The primary outcome data consisted of responses to a survey (see Table 2) administered to the patients and their nurses. The survey was administered on the day of or the day prior to a patient’s discharge from the hospital for patients in the full-access and no-access groups. The surveys were administered either to patients in the noddle group when the patients stopped needing the Voxello technology or prior to discharge. The survey items were composed of statements that were to be evaluated on a 5-point Likert scale (*strongly agree* to *strongly disagree*). Additionally, a text box was provided for open-ended responses. For the no- and full-access groups, the surveys include items related to nurse-call access and the ability to communicate about pain or care (CORE items). For the noddle group, the surveys included the CORE items and items related to the implementation of the noddle and the noddle-chat speech-generating application (implementation items). To ensure that respondents would attend to each item, the survey items were presented either as positive or negative statements (e.g., “I was able to…,” “I wasn’t able to…”). Basic nonidentifying demographic information about the patients in each of the groups was also collected. Nurses who cared for patients in the noddle group were also asked to complete a survey assessing their perceptions of the patients’ ability to summon their nurses and effectively communicate.

### Baseline Comparisons

There were some significant group differences in the demographic data (see Table 1, age and race/ethnicity). However, neither of these variables were significant predictors of the survey data. Therefore, we present the unadjusted results below.

### Number of Cases Analyzed

The survey data from 51 full-access controls, 49 no-access controls, and 36 noddle participants were included in the analyses. Additionally, surveys were obtained from 30 nurses who worked with noddle patients.

### Data Processing

Prior to analysis, the responses to the survey items that were stated in the negative (e.g., “I was not able to…”) were inverted to correspond to the positive form (e.g., “I was able to…”). In addition to examining the responses to individual survey items, we computed a composite score for each subject in the full-access, no-access, and noddle groups’ responses to the CORE items. We then used those composite scores to determine whether there were significant differences in the responses of the three subject groups.

### Outcomes

#### Research Question 1

We predicted that the perceptions of patients who are able to use the nurse call system and communicate would be significantly more positive than patients who are unable to access the nurse call system and unable to communicate with their caregivers.

##### Overall test.

The composite scores were examined between the full-access control, no-access control, and noddle groups using one-way analysis of variance (ANOVA) models. There was a significant main effect of group, *F*(2, 133) = 93.03, *p* < .0001, η^2^ = .58. Post hoc Tukey-adjusted comparisons (see Table 3) revealed significant differences between all three groups. Specifically, the full-access group (*M* = 9.96, *SD* = 2.73) responded more positively than the noddle group (*M* = 13.75, *SD* = 3.25) and the no-access group (*M* = 18.22, *SD* = 3.16). The noddle group rated their experiences more favorably than the no-access control group.

**Figure 3.**
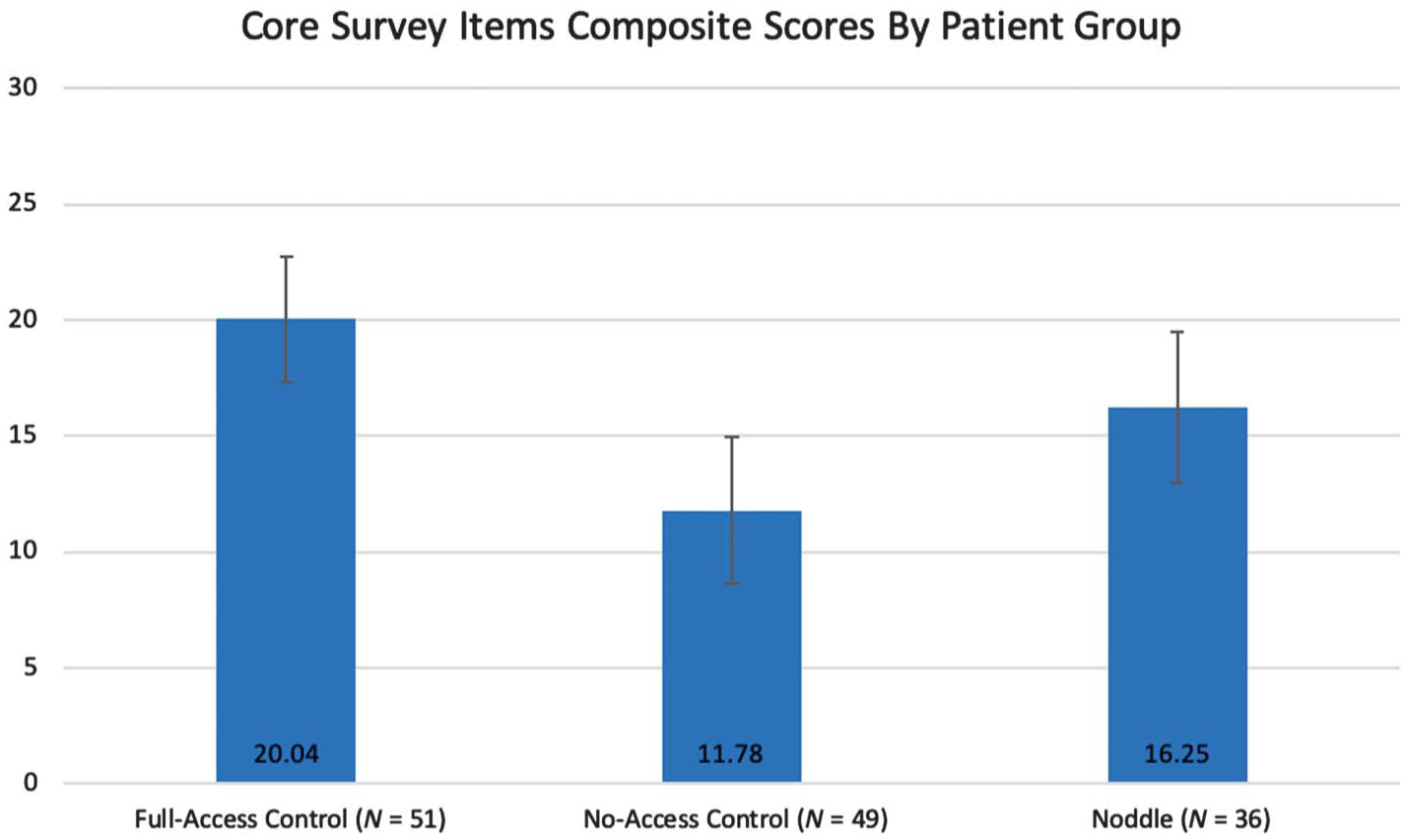
Comparison of composite scores by patient group.

Figure 3 presents the mean composite scores for the three patient groups. A higher score is indicative of more positive perceptions of access to the nurse call and ability to communicate.

**Figure 4.**
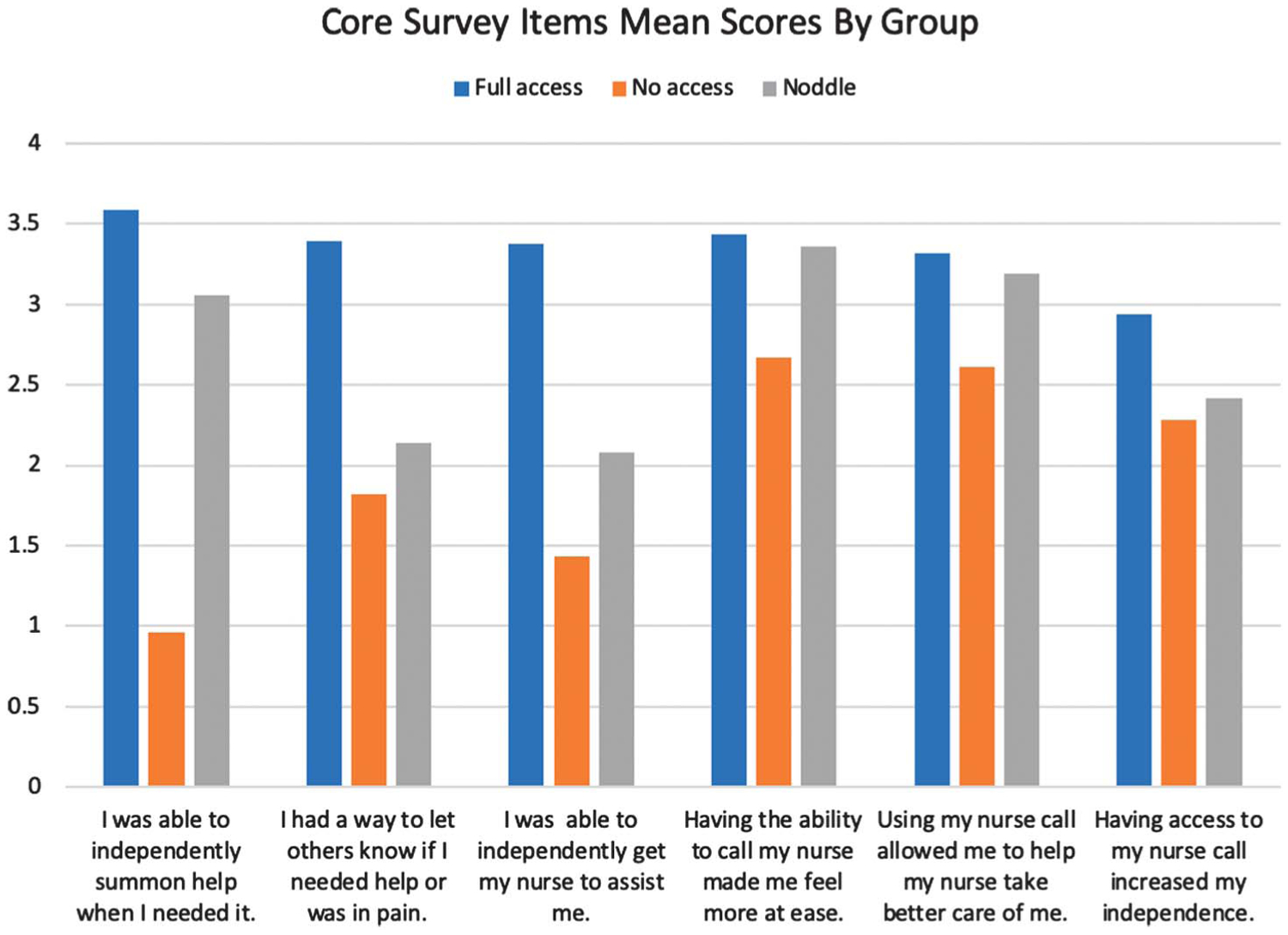
By-group comparison of responses by survey item.

Figure 4 presents the scaled scores by survey item and by group. A higher score is indicative of more positive response. The no-access group had lower mean responses than the full-access group, with the largest differences seen for the first three items. The responses on the first item (“I was able to independently summon help when I needed it”) showed the largest difference of the full-access group and the no-access group. By contrast, the responses of the noddle group resembled those of the full-access group on that item and on the fourth and fifth items (“Having the ability to summon my nurse made me feel more at ease“ and “Using my nurse call allowed my nurse to take better care of me”).

#### Research Question 2

If the intervention provided access to the nurse call and supported communication, it would be perceived favorably by patients and their nurses.

##### Patients’ perception of the noddle.

**Figure 5.**
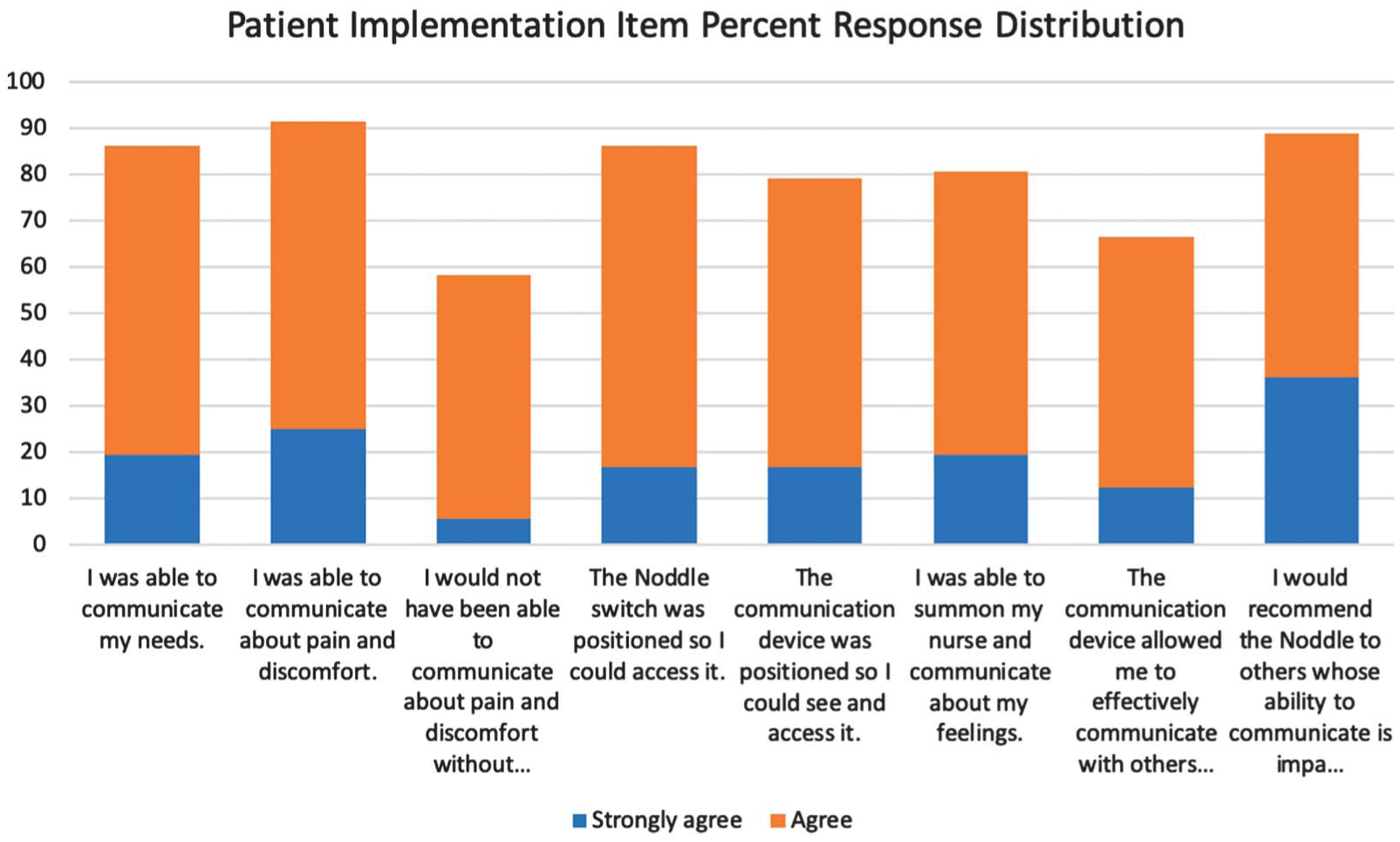
Distribution of responses for patient implementation items.

Figure 5 presents the frequency distribution of the patient responses for the implementation survey items for the patients who were in the noddle group. The majority of the patients in the noddle group indicated agreement (*agree* and *strongly agree*) on all of the items. Item 3 stands out with the lowest cumulative agreement (i.e., “I would not have been able to communicate about pain and discomfort without the device.”). This result might reflect that many patients can express a basic level of pain and discomfort with facial expressions, crying, or moaning.

The noddle patients’ responses to the items about positioning of the devices (the fourth and fifth items in Figure 5) and the implementation of the device were positively correlated (Pearson *r* = .59, *p* < .0032). Furthermore, patients who responded favorably on the implementation items tended to report that they would recommend the use of the technology for other patients with impaired communication (Pearson *r* = .53, *p* < .0083).

##### Nurses’ perception of the noddle.

**Figure 6.**
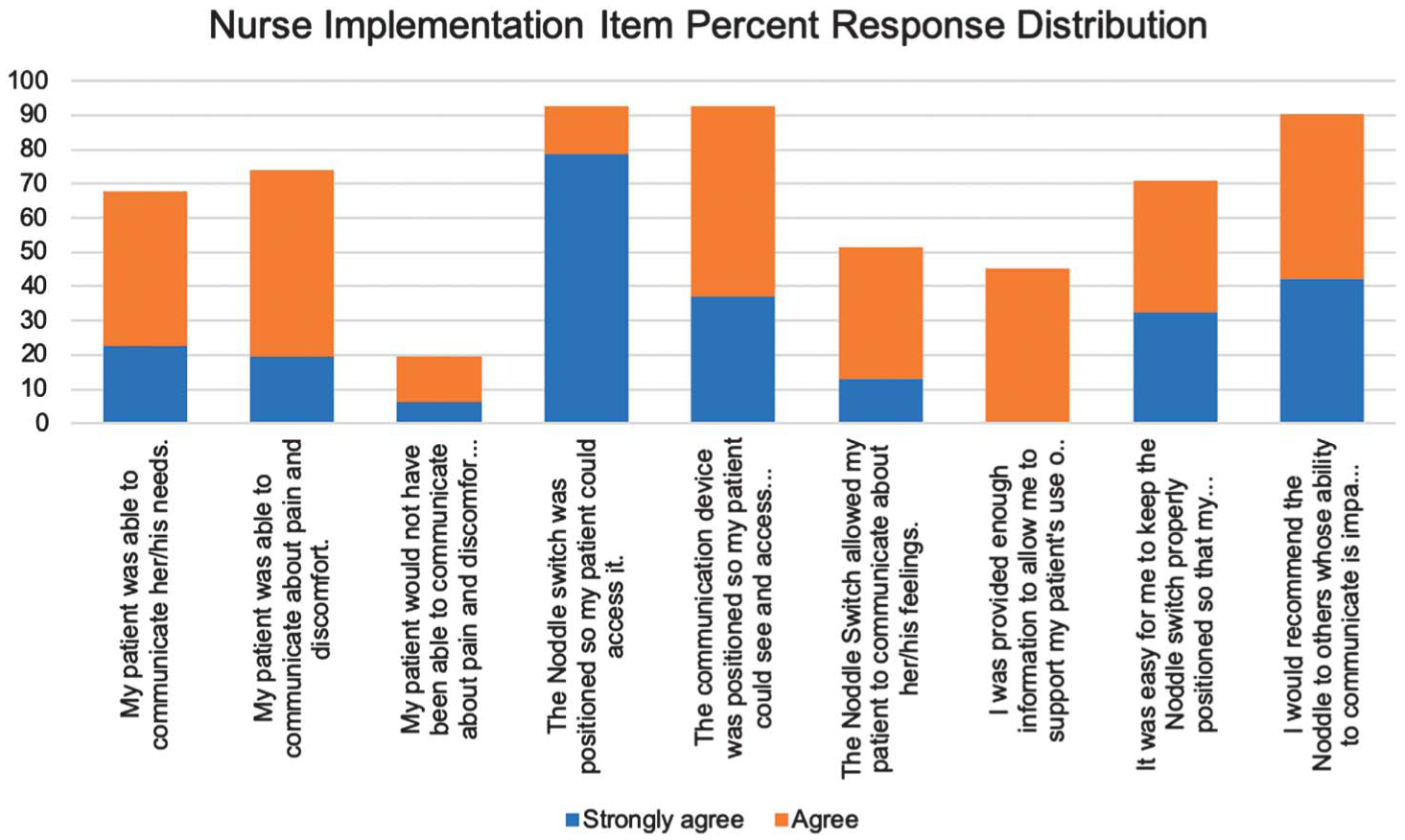
Distribution of responses for nurse implementation items.

As part of the clinical trial of the Voxello technology, nurses working with patients in the study were asked to complete a voluntary survey to assess their perceptions of the patients’ ability to summon them and effectively communicate. Figure 6 presents the frequency distribution for the nurses’ responses for the implementation survey.

There was markedly lower agreement on the third item, “My patient would not have been able to communicate about pain and discomfort without the device.” This reflects the likelihood that nurses can perceive patient distress from their facial expressions and other indications of distress (e.g., vital signs) independent of the patients’ ability to verbalize that they are in pain.

Nurses were less positive about the amount of information that they received about using the noddle (the seventh item in Figure 6), though the nurses were more positive about the ease of keeping the noddle positioned so that the patients could effectively use it (the eighth item in Figure 6). Patients who received the noddle, as well as their nurses, indicated that they would recommend the noddle to other hospitalized patients who faced communication barriers in the hospital (see Figures 5 and 6).

## Discussion

This study examined patients’ and nurses’ perceptions of the patients’ ease of summoning help and effectively communicating about their needs. Specifically, the study compared the perceptions across three groups of patients, who varied as a function of their ability to access the nurse call and to communicate independently. The patients in all three groups were asked to respond to survey items that probed both how the patients felt about their ability to summon help and their ability to communicate with their nurses. Patients in the noddle group and their nurses were also asked about how easy it was to implement the technology.

### Perceptions of Access and Communication

The data revealed significant differences between all three patient groups. Patients with full, independent access to the call system and ability to communicate with nurses responded significantly more positively than patients with no access. Patients in the noddle group, who were provided an AAC system, reported significantly more positive experiences than the no-access group. However, the noddle group’s perceptions were still less favorable than those of the full-access group. It is worth noting that the difference between groups seen in the core composite scores was not evenly reflected in the responses to individual core survey items. Specifically, the no-access and noddle groups both showed lower scores on Items 2 and 3 of the survey (“I had a way to let others know if I need help or was in pain“ and “I was able to independently get my nurse to assist me”; see Figure 4). Nevertheless, it is worth noting that the noddle group patients provided more positive responses compared to the no-access group on all of the survey items.

### Perceptions of the Implementation

The patients in the noddle group were also asked to evaluate the impact of being provided AAC technology that made it possible for them to access the nurse call system and to communicate with their nurses. Overall, the patients rated the noddle implementation favorably—the majority of the responses to each survey item were “agree” or “strongly agree.” Given the diversity in the admitting condition of the patient population and the fact that they were in either the intensive care units or the step-down units, it is not surprising that their experiences would vary to some extent. Differences in the duration of intubation, dependence on ventilatory support, and sedation protocols may also have contributed to differences in their experiences and perceptions of their ability to summon help and effectively communicate.

To ensure that the AAC technology was being implemented with fidelity, patients, nurses, and family members were provided instruction on use of the assistive technology. Nevertheless, changes in the patients’ state might have impacted recall and their ability to use the technology. Given the number of nurses who interacted with each patient in the noddle group, it was difficult to ensure consistency across nursing shifts. In addition to having nurses who are equally well trained, it was difficult to have informed hand-off at shift changes or when a patient moved to another unit. A key to the successful use of any assistive technology is the extent to which the devices are properly positioned where the patients can access them and effectively use them. Nurses were positive about their ability to make the technology accessible to their patients, but they were less positive about having received sufficient training (see Figure 6). Nevertheless, 90% of the nurses strongly agreed or agreed that they would recommend using the technology with other patients who faced access and communication barriers.

The data revealed that the positioning of the noddle and the noddle-chat tablet could serve as a proxy for the AAC implementation fidelity. The significant positive correlation between the survey items related to positioning of the devices and the items that probed the ability to summon help and effectively communicate supports the importance of ensuring that the technology is properly positioned so that patients can effectively use the technology. Furthermore, patients who expressed more favorable perceptions about their ability to communicate also tended to indicate that they would recommend the noddle to others. These findings illustrate the link between implementation fidelity, patient communication experience, and satisfaction.

### Clinical Applications and Future Directions

Unlike a laboratory experiment or a therapy efficacy study in which the treatment is provided by highly trained research assistants or clinicians, an implementation study such as the current study is limited by both the variability in patients that comes from having to use a convenience sample and the large number of nurses and other caregivers who must implement the technology.

The process of introducing any form of AAC into the standard of care in an acute care setting is a developmental one (Costello, Santiago, & Blackstone, 2015; Holden, 2017; Hurtig, Nilsen, Happ, & Blackstone, 2015). The goal is to establish an institutional “culture of communication” that recognizes the barriers to communication faced by patients and the need to embrace a range of strategies to overcome those barriers. In addition to physically based communication barriers, a significant number of patients with limited English proficiency are unable to effectively communicate with their caregivers and are also at a higher risk of experiencing preventable AEs (Bartlett et al., 2008; Cohen et al., 2005; Hurtig et al., 2018). While hospitals are required to provide access to interpreter services (The Joint Commission, 2010), they cannot realistically be involved in all of the patients’ bedside care interactions. Hurtig, Czerniejewski, Bohnenkamp, and Na (2013) suggested that there is a role for implementing AAC strategies in addressing the communication needs of patients with limited English proficiency. When hospitals can use similar tools to meet the needs of patients with CCN regardless of whether they face a physical or linguistic barrier, the burdens associated with training nurses can be reduced (Blackstone, Garrett, & Hasselkus, 2011; Blackstone & Pressman, 2016; Blackstone, Ruschke, Wilson-Stronks, & Lee, 2011).

Best practice in traditional AAC implementation for individuals with developmental and acquired disabilities has always recognized that, to be effective, communication partners must be considered. To that end, it is important to understand what the communication partners’ needs are and to structure the intervention with those needs in mind. No matter how simple and transparent AAC strategies may appear to the AAC specialist, the communication partners of the individual who faces a communication barrier will need to understand how the pragmatics of an interaction are altered by the need to use AAC strategies. Therefore, the approach to training communication partners must be implemented using a dynamic assessment model.

While the patient and nurse training used in the study was informed by training used in other settings in which assistive technology is introduced to users and their support personnel (e.g., parents, teachers, family members, and peers), it is evident from the perspective of both the patients and the nurses that more will be needed to achieve a higher fidelity of implementation. Part of the problem may stem from the temporal dissociation between when training on the use of the technology is provided and when the nurses and patients need to use the technology. Feedback from nurses and nurse managers indicated that there is a greater need for just-in-time training. This training would help nurses directly and help them provide continued support to their patients who might need reminders about how to use the AAC system. Given this feedback, shorter and component training materials have been developed. These include brief video clips that nurses can access at the bedside to support setting up the technology and to demonstrate the use of the technology to the patient. These materials are now available to the Voalte phones that nurses use to respond to their patients, communicate with other members of the health care team, and access a range of instructional materials.

Overall, this study suggests that AAC can be successfully used in acute care settings to help patients overcome access and communication barriers. However, simply making the technology available at the bedside will not be sufficient. For patients who can use the standard nurse call pendant, their ability to summon help will depend on nurses making sure that the pendant works and is within reach. Likewise, for patients who need AAC (either low or high tech), nurses will need to be vigilant that the technology is accessible to the patient. While rehabilitative services personnel (speech-language pathology and occupational therapy) have historically been the health care professionals associated with the delivery of AAC services (American Speech-Language-Hearing Association, 2016, n.d.), they are not the only professionals with an interest in enhancing patient–provider communication (Altschuler & Happ, 2019). Given the fact that nurses are typically the patients’ primary communication partners, it is critical that there be support and buy-in from nursing administrators and the medical directors of the inpatient units.

From the patient perspective, being able to summon help and communicate with caregivers allows them to effectively make their needs known and more actively participate in their care. Overcoming communication barriers should reduce anxiety and the risk of experiencing preventable AEs, resulting in increased satisfaction with the care provided. Anecdotally, none of the patients in the noddle group experienced a preventable AE during their hospitalization. From the nursing perspective, the inability to know what a patient needs and wants can be very stressful. Being able to effectively communicate with their patients makes it easier to care for them and reduces the nurse’s stress.

## Summary

This study revealed significant differences between patients who could and could not summon their nurses and communicate with them. Providing access to patients who could not access the nurse call and communicate significantly improved those patients’ perceptions. The patients receiving the AAC intervention and their nurses responded positively to the intervention and indicated that they would recommend use of the technology for other patients facing communication barriers. To increase implementation fidelity and improve outcomes, additional efforts will need to be directed toward optimizing how nurses are trained to use the technology and support their patients’ use of the technology.

## Supplementary Material

Demo Video

## Figures and Tables

**Table 1.** Demographic data for the noddle and control groups.

	Group			Group comparisons
Variable	Full-access control (*n* = 51), %	No-access control (*n* = 49), %	Noddle (*n* = 36), %	Wilcoxon scores Kruskal-Wallis test
Age (years)				*p* > .02
≤ 25	8	4	0	
26–45	8	16	30	
46–65	39	35	53	
66–85	35	41	17	
≥ 86	10	4	0	
Education				*p* < .23
< High school	8	0	6	
High school	47	47	36	
Community college	29	37	25	
College	10	14	22	
Graduate/professional	6	2	11	
Race and ethnicity				*p* < .01
White	96	100	80	
African American	2	0	17	
Hispanic/Latino	0	0	0	
Native American	0	0	0	
Asian/Pacific Islander	2	0	3	
Other	0	0	0	
Gender	49			*p* < .06
Female		53	28	
Male	51	47	72	

**Table 2.** Survey items.

Patient core survey (all patient groups)	Patient implementation survey (noodle group)	Nurse survey (noddle group)
I was able to independently summon help when I needed it.	I was able to communicate my needs.	My patient was able to communicate her/his needs.
I had a way to let others know if I needed help or was in pain.	I was able to communicate about pain and discomfort.	My patient was able to communicate about pain and discomfort.
I was able to independently get my nurse to assist me.	I would not have been able to communicate about pain and discomfort without the noddle switch.	My patient would not have been able to communicate about pain and discomfort without the noddle switch.
Having the ability to all my nurse made me feel more at ease.	The noddle switch was positioned so I could access it.	The noddle switch was positioned so my patient could access it.
Using the nurse call allowed me to help my nurse to take better care of me.	The communication device was positioned so I could see it and access it.	The communication device was positioned so my patient could see and access it.
Having access to my nurse call did not increase my independence.	I was able to summon my nurse and communicate about my feelings.	The noddle switch allowed my patient to communicate about her/his feelings.
	The communication device allowed me to effectively communicate with others.	I was provided enough information to allow me to support my patient’s use of the noddle switch.
	I would recommend the noddle to others whose ability to communicate is impaired while in the hospital.	It was easy for me to keep the noddle switch properly positioned so that my patient could access it.
		I would recommend the noddle to others whose ability to communicate is impaired while in the hospital.

**Table 3.** Tukey studentized range test

Group comparison	Absolute difference between means	Simultaneous 95% confidence intervals		Significance level
Full access-no access	8.2637	−9.7004	−6.8270	*
Full access-noddle	3.7892	−5.3527	−2.2258	*
Noddle-no access	4.4745	−6.0511	−2.8979	*

* Comparisons significant at the .05 level.
